# Endoscopic palliation of advanced esophageal cancer

**Published:** 2015

**Authors:** A Mocanu, R Bârla, P Hoara, S Constantinoiu

**Affiliations:** * Surgery Clinic, “Sf. Maria” Clinical Hospital, Bucharest , Romania

**Keywords:** advanced esophageal cancer, palliation, endoscopy, combined therapy

## Abstract

Esophageal cancer represents one of the most aggressive digestive tumors, with a survival rate at 5 years of only 10%.

Globally, during the last three decades, there has been an increasing incidence of the esophageal cancer, approx. 400,000 new esophageal cancers being currently diagnosed annually. This represents the eighth leading cause of cancer incidence and the sixth leading cause of cancer death overall. Taking into account the population’s global aging and thus, the increase in the number of patients who will not bear surgery, PCT and radiation, or the fact that they do not want it especially because of deficiencies and associated pathology, the endoscopic ablative techniques with palliation purposes represent the alternative.

If we refer to the Western Europe countries and North America, we notice an increase of esophageal adenocarcinoma rate versus squamous cancer. As for the Asian region, referring in particular to China and Japan, 9 out of 10 esophageal cancers are squamous cell carcinomas.

For at least half of the patients with EC (esophageal cancer) there is no hope of healing because of the advanced regional malignant invasion (T3-4, N+, M+) with no chemo and radiotherapy response, poor preoperative patients’ conditions or systemic metastasis. The low life expectancy does not justify the risky medical procedures, the goal of the therapy consisting in the improvement of the quality of life by eliminating dysphagia (reestablishing oral feeding) which represents the most common complication of EC, the respiratory tract complication caused by eso-tracheal fistulas or by eliminating chest pain. To treat dysphagia, which is the main target of palliation, combined methods like endoscopic, chemo and radio-therapy, can be used, each one with indications, benefits and risks.

**Abbreviations:** SEPS = self expanding plastic stent, SREMS = self expanding metal stent, EBRT = Endoscopic brachy radiotherapy, EUS = Ultra sound endoscopy, CT = Computer tomograph, UGE = Upper gastro endoscopy, PET-CT = Positron Emission Tomography, APC = argon plasma coagulation, PDT = photo dynamic therapy, PCT = Poli-chemotherapy, RT = Radio-therapy

## Introduction

There are several interventional endoscopy techniques designed to eliminate dysphagia, known as: 1-mechanical methods (dilatations and esophageal stents) and, 2-ablation methods by using chemic or physical techniques. 

**Esophageal stents**

Esophageal stents are important tools for palliative treatment of inoperable esophageal malignancies. With the development of multiple self-expandable stents, there are now several therapeutic options for the management of benign and malignant esophageal diseases. 

Historically, esophageal stents have been used to palliate patients with dysphagia or obstruction caused by a malignancy [**1**]. However, these rigid plastic prostheses have been associated with high complication and morbidity rates [**2**]. Currently, the esophageal stents are made from metal alloy compounds and durable polymers, and these stents are used for the treatment of a variety of benign and malignant esophageal conditions.

With the recent development of self-expanding plastic stents (SEPS) and self-expanding metal stents (SEMS), stent placement for the esophageal pathologies can be safe and cost-effective.

**Types of Esophageal Stents**

**Fig. 1:**
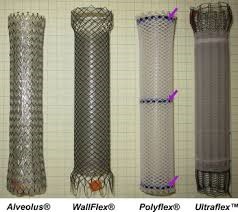
Partially covered SEMS

A variety of SEPS and SEMS are currently available in the United States, with 3 types regarding the exterior cover: uncovered, partially covered and fully covered. The original esophageal SEMS were uncovered, with no synthetic material covering the metal mesh. However, a variety of covering materials (most commonly polytetrafluoroethylene) have been developed due to complications of tumor and granulation tissue ingrowth. Fully covered stents do not have any exposed bare metal, being more prone to stent migration. Partially covered SEMS have a small portion of exposed bare metal at the proximal and distal ends to allow the embedding into the esophageal wall, which helps to prevent migration [**3**] (**Fig. 1**).

**Difficulties in Esophageal Stent Placement**

The placement of esophageal stents may be associated with several challenges depending on the location of strictures or tumors in the esophagus. Because a stent must be long enough to bridge a stricture and extend 2–4 cm beyond each end, a stricture proximally or distally located can be difficult to stent properly. Strictures that have narrow or tortuous lumens present another challenge because the luminal diameter must allow the passage of the endoscope [**4**].

**High-Grade Strictures**

**Fig. 2:**
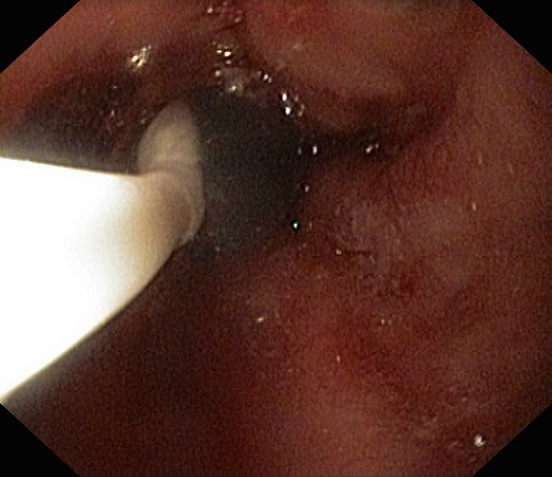
Dilator

If a stricture is very tight or difficult to traverse with a standard endoscope, there are currently several ways to bypass it [**5**]. The first option is to use a dilator (**Fig. 2**). Currently, there are 3 types of available dilators. Savary-Gilliard dilators and TTS balloon dilators are currently the most commonly used ones [**5**]. Another method is using a stent with a smaller diameter. Pediatric endoscopes, which are usually small in diameter (5–8 mm), can also be used [**6**].

**Upper Esophageal and Cervical Esophageal Strictures**

Traditionally, strictures close to the upper esophageal sphincter (UES) have been considered more difficult to manage. In the past, the use of stents was limited by the patient’s complaints of chest pain and globus sensation, as well as complications such as perforation, proximal migration, and aspiration pneumonia [**7**,**8**]. However, studies have recently demonstrated the effectiveness and safety of stent placement for the palliation of dysphagia and sealing of fistulae in patients with strictures close to the UES [**9**]. Verschuur and associates examined 104 patients with malignant strictures within 8 cm of the UES; the researchers achieved technical success in 96% of the patients, an average improvement in the dysphagia score from 3 to 1, and a fistula-sealing rate of 79% [**16**].

**Distal Esophageal Strictures**

Distal esophageal strictures still present a significant challenge because stent placement across the gastroesophageal junction can lead to gastroesophageal reflux disease and aspiration. In an attempt to remedy these problems, stents with antireflux mechanisms have been developed. A randomized controlled trial by Laasch confirmed these findings, as reflux was seen in only 12% of the patients (3/ 25) who received an antireflux Esophageal Z-Stent, compared to 96% of patients (24/ 25) who received a standard open stent (P<.001) [**10**].

However, recent RCTs have not reproduced these findings [**11**,**12**]. Blomberg and associates studied 65 patients who received an antireflux Esophageal Z-Stent or a standard stent [**11**]. No differences were found in health-related quality of life due to reflux. Given these equivocal data, larger studies and/ or improved study designs are necessary to determine whether antireflux valves are effective.

**Management of Malignant Esophageal Stenosis**

Despite the advances in the diagnosis, staging, preoperative and perioperative care of patients with esophageal cancer, the 5-year survival rate of these patients remains less than 15%, and chemotherapy has shown limited survival benefit [**13**,**14**]. Therefore, patients with incurable esophageal and other non-luminal malignancies of the head and neck often require palliation for dysphagia and/ or trachea-esophageal fistulae.

**Self-Expanding Metal Stents (SEMS)**

Since the introduction of SEMS 20 years ago, these stents have been shown to be safer and more cost-effective than the plastic esophageal prostheses previously used [**36**]. In a retrospective study of 153 patients, Eickhoff found comparable rates of survival, recurrent dysphagia, and improvement in dysphagia scores between SEMS and SEPS; however, SEMS had a much lower complication rate than SEPS (9% vs. 22%, respectively) [**15**]. Currently, SEMS and SEPS have become the standard of treatment for malignant esophageal strictures and fistulae.

As previously discussed, covered stents resist tumor ingrowth because they do not have an uncovered region that embeds into tissue; however, covered stents are also more susceptible to stent migration. In a retrospective study of 152 patients who received either a covered or uncovered stent, Saranovic and associates found that covered stents were associated with more migration (10% vs. 0%) but less tumor ingrowth (53% vs. 100%) and less restenosis with recurrent dysphagia (8% vs. 37%) than uncovered stents [**38**]. Fully covered stents also offer the advantage of being completely removable.

Although the use of SEMS to treat upper esophageal cancer is widely accepted, their use for treating cancer closer to the UES is controversial because of the perceived increased risk of complications such as perforation, migration, pain, and patient intolerance. A recent study showed that the mean dysphagia score decreased by the same amount in both patients with upper esophageal cancer and patients with lower esophageal cancer who were treated with the same types of SEMS [**16**]. In addition, there were no statistically significant differences in early or late complications or median survival rates, showing that SEMS effectively treated both proximal and distal cancers of the esophagus.

**Self-Expanding Plastic Stents (SEPS)**

In the early 2000s, SEPS emerged as an alternative to SEMS. Costamagna described the use of plastic Polyflex Esophageal Stents for the management of 16 patients with inoperable esophageal strictures [**17**]. After stent placement, patients experienced significant improvement in dysphagia; complications included stent migration (in 2 patients) and repeated interventions (in 4 patients). Szegedi and associates conducted a large study in which SEPS were placed for palliation of malignant dysphagia in 66 patients; the researchers reported a dysphagia score improvement, a migration rate of 4.5%, and no tumor ingrowth during a mean follow-up period of 129 days [**18**].

**Biodegradable Stents**

Biodegradable stents have recently been developed to avoid the complications of tissue ingrowth and migration and decrease the need of stent removal. The use of biodegradable stents remains problematic due to complications of migration, stricture recurrence, and tissue ingrowth; 1 case study even reported the development of a trachea-esophageal fistula [**19**]. These stents also present new challenges: In 1 case report, the biodegradable stent mesh collapsed inside the esophageal lumen, preventing the passage of a standard endoscope [**20**].

The complications associated with esophageal stents are generally classified as either early or delayed [**4**]. Early complications occur immediately or within 2–4 weeks and include chest pain, fever, bleeding, gastro-esophageal reflux disease, perforation, and stent migration [**21**].

In 1 study, early complications were reported in up to 32% of patients, with stent migration being the most common complaint [**22**]. Prolonged chest pain was reported in 12–14% of the cases, while rates of direct perforation were lower. A small amount of bleeding is relatively common after stent placement; more severe bleeding is rare, occurring in 1% of the cases in 1 study [**23**]. Among both early and delayed complications, stent migration is the most common complication, occurring at a frequency of 7–75% [**24**].

**Fig. 3:**
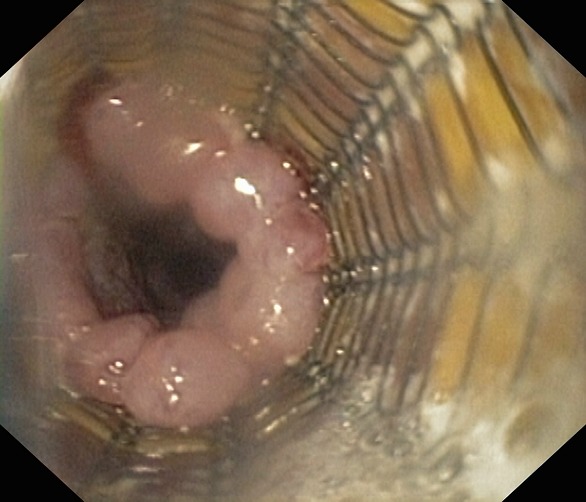
Delayed complication: tumor ingrowth

**Fig. 4:**
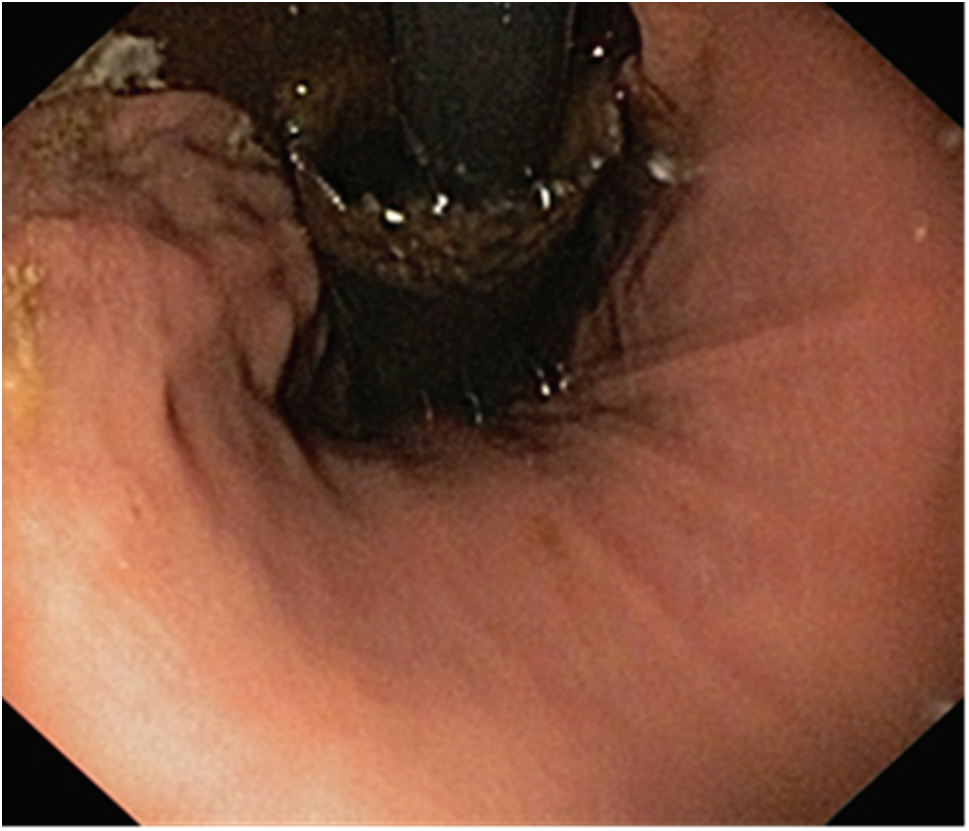
Tumor ingrowth

Delayed complications are more common than the early ones and are defined as complications that occur at least 2–4 weeks after the placement of a stent. These complications include tumor ingrowth (**Fig. 3**,**4**), stent migration, stent occlusion, development of esophageal fistulae, and recurrence of strictures [**25**]. 

Delayed complications have been reported in 53–65% of the patients, with a reintervention rate of up to 50% [**26**,**4**]. In a study of 133 patients who underwent a placement of SEMS for the treatment of malignant strictures, Homann and colleagues reported an overall delayed complication rate of 53.4% (71/ 133 patients) [**6**]. Recurrent dysphagia was caused by tumor ingrowth (22%), bolus obstruction (21%), stent migration (9%), or esophageal fistulae (9%). In another study, approximately 0.5–2% of the patients died as a direct result of esophageal stents.

Esophageal stents remain important tools for the palliative treatment of inoperable esophageal cancers. With the development of multiple SEPS and SEMS, there are now several therapeutic options for the management of benign and malignant esophageal diseases. The minimally invasive approach of esophageal stenting has improved the quality of life of these patients, who would otherwise face a possibly morbid surgical procedure or who may have limited treatment options because of multiple comorbidities. In the future, innovations such as biodegradable stents might improve stent patency and mitigate stent-related complications.

**Dilatations**

**Fig. 5:**
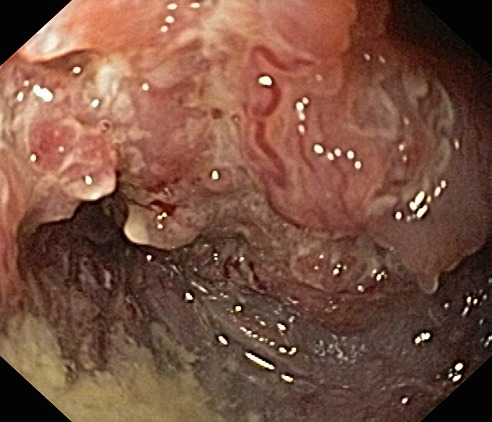
Complex strictures

The main indication for esophageal dilation is to relieve benign or malignant dysphagia [**27**,**28**]. The endoscopic dilation of malignant strictures is also performed to facilitate the completion of endoscopic procedures, such as EUS (endoscopic ultrasound) tumor staging, to permit the placement of esophageal stents or to place a PEG for feeding purposes [**29**,**30**]. Esophageal strictures can be structurally categorized into two groups: complex and simple. Complex strictures are those that are asymmetric, irregular or angulated with the diameter of less than 12 mm (**Fig. 5**).

**Fig. 6:**
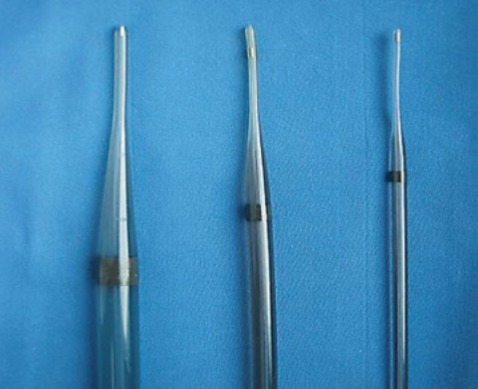
Bougies used to perform the esophageal dilation

Simple strictures are symmetric or concentric with a diameter of 12 mm that easily allow the passage of a diagnostic upper endoscope [**31**-**33**]. The esophageal dilation is currently performed by using either bougies or balloons [**31**,**34**-**36**] (**Fig. 6**). 

Currently two main types of bougies are used: mercury or tungsten-filled bougies (Maloney or Hurst) and over-the-wire (OTW) polyvinyl bougies (Savary-Gilliard® Or American, Wilson-Cook Medical, Inc., Winston-Salem, N.C., USA). The risk of esophageal perforation may be higher with a blind passage of the Maloney dilators than with OTW Savary or TTS (through-the-scope) balloons, particularly in patients with a large hiatal hernia, a tortuous esophagus, or those with complex strictures [**31**-**34**].

**Fig. 7:**
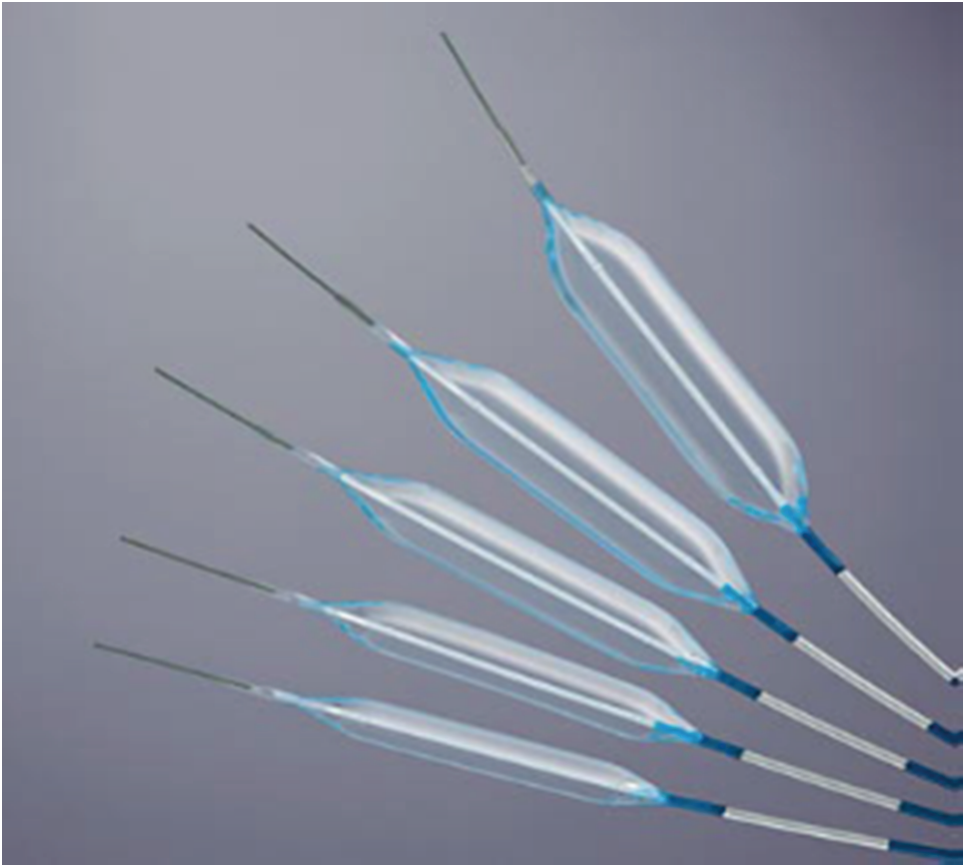
Esophageal balloon dilators

Various types of balloons are available to dilate the esophagus. Balloons for esophageal stricture dilation come in various shapes and sizes (**Fig. 7**).

Because these balloons can be advanced through the accessory channel of the endoscope, they are also referred to as through-the-scope (TTS) balloons [**37**]. Although the choice of dilatation device is left to the endoscopist preference, dilations in patients with tumors are mainly performed with Savary dilators following the conventional technique, by using incremental diameters of the bougies, but no more than three per session “rule of-three”, and always under fluoroscopic monitoring. Although this rule does not apply to balloon dilators, a recent study suggested that the inflation of a single large-diameter dilator (>15 mm) or incremental dilation of >3 mm may be safe in simple esophageal strictures [**38**]. Actually, most balloons allow a three-step inflation process, practically paralleling the “rule of-three”.

**Complications**

Regardless of the specific method of dilation, the early improvement in the ability of swallowing is achieved in virtually all the patients. 

The esophageal perforation is the major complication associated with endoscopic dilation [**34**,**39**,**40**]. The perforation rate after dilation for esophageal strictures was reported to be of 0.1–1% [**41**]. A United Kingdom regional audit reported an overall perforation rate of 2.6% with a mortality of 1% [**40**]. In that study, the perforation was less common following the dilatation of benign strictures (1.1%) than following the dilatation of malignant strictures (6.4%) [**42**].

**Ablation methods**

The endoscopic ablation therapy uses a number of chemical and phisical methods to complete the local distruction of the malignant tisue; unfortunately, these technologies can only be used for short tumors. The goal is the selective distruction of the neoplasia. Nevertheless, it is as complete is the enndoscopic thumorectomy, as high is the rate of complications. The following can be used:

-Photodynamic therapy (PDT)

-lasertherapy

-argon ablation (APC)

-brachytherapy (EBRT)

**Photodynamic therapy**

PDT - represents a two-stage ablation technique which does not use temperature, yet it relies on oral or i.v. administering a photosensitive substance with tropism for the neoplastic tissue or BE-type tissue [**43**]. The further stimulation of the substance with a beam of light of a precise frequency (630 nm) leads, after a photochemical reaction, to the release of oxygen radicals that will cause the target-cell destruction.

The substance used is a sodium salt of porphyrin, the commercial preparation used in the US as Photofrin. In Europe it is used as a photosensitizing substance 5-aminolevulinic acid (5-ALA). There are currently no studies comparing the efficacy of the two substances, but both remain at a fairly high price. Apparently, using 5-ALA would predispose slightly less at strictures as late complications of the method. Once stimulated, the photosensitive substance, the tissue necrosis process takes about 8 to 10 days [**44**].

Most of the use of PDT has been historical [**45**-**49**], and other modalities have supplanted its application [**50**-**58**], in part because of its side effect profile (pain, esophageal stricturing, perforation, and cutaneous photosensitivity) and cost of the drug. 

A Korean study, in turn, demonstrated a significant reduction in dysphagia in 90% of 20 patients treated de novo with PDT. The rate of esophageal stricture was of 10% and the median survival approximated 7.0 months [**59**,**60**].

A study reviewed 640 patients with esophageal cancer treated at the Medical University in Graz-Austria, between 1999 and 2009 [**61**]. Two hundred and fifty patients (39.1%) were treated with palliative intent by using a variety of techniques to include dilation, stenting, brachytherapy, chemotherapy, external irradiation, and PDT. Palliation with PDT was ultimately undertaken in 171 patients, 118 as initial therapy. The median survival in the latter group was of 50.9 months compared with 17.3 months if the other therapies were initially used (P = 0.012), and the overall survival of the palliative group was of 34 months. The authors suggested that the prolonged survival in the patients initially treated with PDT was more likely related to a secondary immune response by T cells activated by the inflammatory necrosis as opposed to the acute local effect of PDT.

Rupinski et al. [**62**] studied 93 patients with malignant dysphagia, who were treated by using three palliative regimens: brachytherapy, PDT, or argon plasma coagulation (APC) alone. The time to first dysphagia recurrence was significantly different between the PDT and brachytherapy groups and those patients treated with APC alone (P = 0.006), but not between the combination groups. Complications were limited to fever in three of the PDT patients, and there was a median survival of 6.2 months with no significant difference between the groups.To date, there is a paucity of studies comparing PDT to techniques (EMR, ESD, radiofrequency ablation, cryotherapy) that have mostly supplanted its application [**63**].

The procedure has proven useful in high-risk patients with de novo malignancy or those who develop recurrent dysphagia following previous RT and PCT or surgery [**64**-**67**].

**Laser therapy**

Greater experience exists with high-energy lasers, particularly the Nd:YAG laser, which causes heating and vaporization of tumor tissue through the delivery of an intense beam of laser light [**68**,**69**]. This causes a burn that is deep enough to effectively and rapidly reconstitute the patency of the esophageal lumen. Most operators use noncontact quartz fibers to deliver the laser energy. The fibers are cooled either by water or by coaxial carbon dioxide (CO2) and are easily passed through the suction channel of standard gastroscopes. The laser beam is aimed directly at the obstructing tumor from a distance of about 1 to 2 cm. With the laser set to the maximum output, 60 to 100 watts of energy is delivered, producing both coagulation and vaporization. Patency (as evidenced by an improvement in the ability to pass the scope and by an improved dysphagia grade) can be achieved immediately in some patients, and on the same day in many patients. Initial studies of esophageal cancer treatment with Nd:YAG laser demonstrated success in palliating dysphagia in 70 to 80 percent of the patients [**68**,**69**]. Patients with short bulky mid-esophageal tumors seemed to achieve the greatest benefit from Nd:YAG laser treatment whereas patients with long (> 8 cm) infiltrating tumors in the upper or lower esophagus did less well [**69**,**70**]. The average duration of the palliation with Nd:YAG laser is estimated at about 4 weeks. In a prospective comparison between Nd:YAG tumor ablation and intubation with an Atkinson tube, Loizou and colleagues demonstrated a comparable success at palliating dysphagia; for patients with tumors crossing the cardia, however, stenting provided superior palliation, with fewer procedures. For the other patients, laser therapy provided a greater ability to eat solid food and was associated with a lower perforation rate [**71**].

**Combined modalities**

Nd:YAG laser therapy and radiotherapy are frequently administered in a concurrent or sequential fashion for pallia¬tion of dysphagia. In a randomized study that compared laser recanalization alone with laser recanalization plus palliative EBRT (30 Gy in 10 fractions), the addition of radiotherapy increased the average interval between laser treatments from 5 to 9 weeks. Others have reported that brachytherapy (intra-luminal radiotherapy) in one or two fractions (total 10–15 Gy at 1 cm from the source) can more than double the inter¬val between laser treatments with minimal morbidity [**72**,**73**].

**Argon plasma coagulation**

Argon plasma coagulation is an ablative endoscopic technique. A form of monopolar electrocautery, APC causes tissue coagulation, desiccation, and destruction via the transfer of energy from the APC probe to the malignant tissue in the form of ionized, electrically conductive argon gas (plasma). The APC probe produces a plasma arc that destroys tissue to a depth of approximately 2–3 mm.

Heindorff et al. [**74**] used APC for palliation in 83 patients with inoperable car¬cinoma of the esophagus or cardia. The esophageal lumen was adequately recanalized in 58% of the patients with one treatment session, and 26% of the patients required two treat-ments. In the other 16% of the patients, treatment failed. Two thirds of the patients needed retreatment at every 3 or 4 weeks until death, with a mean of 6 treatment sessions per patient, and one third of the patients subsequently required the place¬ment of an esophageal stent. Although the success rate of APC in this study compares favorably with other palliative modalities for malignant dysphagia, the high frequency of additional treatments emphasizes the need for a further study of this technique before it can be fully endorsed.

**Brachyterapy**

Brachytherapy was used for the paliation of esophageal cancer for several years and several reports have proved its efficacy. The efficacy of brachitherapy was shown by Jager et al. who reported a series of 88 patients treated with a single fraction of brachyT [**75**]. Seven patients were previously treated by EBRT. The first 51 patients were treated with a medium-dose rate with a treatement range between 2,5 and 5 hours, while 37 patients were treated with HDR. Dysphagia improved in 67% of the evaluated patients. 13% of the patients reported no changes of dysphagia and 20% registered a progression. Fistula occurred in 5 (6%) patients and bleeding only in one.

Sharma et al. treated 58 patients with HDR endoluminal brachytherapy [**76**]. Fifteen patients were previously treated with EBRT in doses of 20 to 30 Gy. The overall improvement of dysphagia was reported in 48% of the patients, 15% developed strictures, 10% ulcerations and 5% fistulas. The median time of stenosis redeveloping was of 4,2 months.

Two studies directly compared the metal stent placement to brachytherapy palliation for advanced esophageal cancer. Homs et al. randomised 209 patients in nine hospitals in Netherlands [**77**]. Patients with tumors greater than 12 cm long or with eso-tracheal fistulas were not included. For patients with brachytherapy, the target volume included was of 2 cm above and below the apparent margins of the tumor and a single dose of 12Gy was delivered to a depth of 1 cm from the source axis. Dysphagia improved faster after stent placement but, after 30 days from the treatment, dysphagia score was similar in the two groups with an improvement of at least 1 grade at 76% of the patients who had stent placement and 73% in patients with brachytherapy. Patients with brachytherapy had more days with no dysphagia (grade 0-1) than those assigned to stent placement (155 vs. 82 P=0,015). The median survival was comparable and medical costs for the two methods were significantly equal. The authors concluded that brachytherapy gave a better long-term relief of dysphagia and was better tolerated. In the second study done in Sweden, by Berquist, on 63 patients, the conclusion was that the stent placement was more effective in the first month but at three months monitoring, brachyterapy offered a better palliation and better quality of life.

## Conclusions

Advanced esophageal cancer continued to be difficult to control and cure, many patients requiring the palliation of their symptoms. The most frequent and significant of these is dysphagia. Endoscopical therapies can be a safe and effective method of producing durable palliation when offered by a methodical, multidisciplinary team of health specialists.
